# Comparing the Quality and Readability of ChatGPT-4-Generated vs. Human-Generated Patient Education Materials for Total Knee Arthroplasty

**DOI:** 10.7759/cureus.86491

**Published:** 2025-06-21

**Authors:** Kirk Lower, Jia Y Lin, Deanne Jenkin, Chantal L Campbell, Ishith Seth, Matthew Morris, Sam Adie

**Affiliations:** 1 Medicine and Surgery, Griffith University, Gold Coast, AUS; 2 Orthopedic Surgery, Gold Coast Health, Gold Coast, AUS; 3 Orthopedic Surgery, St. George and Sutherland Centre for Clinical Orthopaedic Research, Sydney, AUS; 4 Medicine and Health, University of New South Wales, Sydney, AUS; 5 Surgery, Gold Coast Health, Gold Coast, AUS; 6 Surgery, Peninsula Health, Melbourne, AUS; 7 Surgery, Monash University, Melbourne, AUS

**Keywords:** arthrosplasty, artificial intelligence, large language models, patient education, total knee replacement

## Abstract

Background

The purpose of this study was to evaluate the potential role of artificial intelligence, specifically ChatGPT-4, in generating patient education materials (PEMs) for total knee arthroplasty (TKA). The aim of our study was to compare the quality and readability of PEMs for TKA generated by ChatGPT-4 with those created by human experts to assess the potential for the use of AI in patient education.

Materials and methods

We assessed the quality and readability of TKA PEMs produced by ChatGPT-4 and five reputable human-generated websites. Readability was compared using Flesch-Kincaid Grade Level and Flesch Reading Ease. The quality of information was compared using the DISCERN criteria.

Results

ChatGPT-4 PEMs demonstrated a significantly higher reading grade level and lower reading ease score compared to human-generated PEMs (<0.001).

Conclusions

The utility of ChatGPT-4 for producing TKA PEMs is promising. Notably, the quality and reliability are as good as human-generated resources. However, it is currently limited by readability issues, leading to a recommendation against its use. Future AI enhancements should prioritize readability to ensure information is more accessible. Effective collaboration between AI developers and healthcare professionals is vital for improving patient education outcomes.

## Introduction

Total knee arthroplasty (TKA) accounts for a significant proportion of orthopedic procedures performed globally each year. The Australian Orthopaedic Association National Joint Replacement Registry estimates that over 880,000 TKAs were performed in Australia between 1999 and 2022 [1]. Additionally, the incidence of TKA for osteoarthritis is expected to grow by 276% by the year 2030 [2]. Compared to the available 2014 National Inpatient Sampling (NIS) figures, the projected total annual United States use for TKA is expected to increase by 182% in 2030 [3].

Concurrently, the internet has become a prevalent medium for disseminating health-related information, attracting over eight million daily users in the United States [4]. Notably, orthopedic patients exhibit significant internet utilization for health education [5]. However, the majority do not share this information with their surgeons [6], highlighting the crucial importance of health literacy.

Health literacy pertains to one’s ability to access, comprehend, and utilize fundamental health information and services to enhance well-being [7]. It stands as an independent predictor of health-related quality of life [5], with reduced health literacy linked to heightened hospitalization rates [8], increased postoperative complications [9], elevated mortality [10], and substantial economic burden, comprising between 3% and 5% of Australian healthcare system costs [11]. Therefore, it is crucial that patient education materials (PEMs) are of sufficient quality and tailored to an appropriate readability level.

Based on the assessment of the reading ability of adult Americans, the US Department of Health and Human Services recommends that health information should be written at or below the sixth‐grade level, which is the level of an 11‐ to 12‐year‐old child with six years of US education [12], while the Institute of Medicine recommends a reading level of sixth‐ to eighth‐grade or below [13].

The evolution of AI-generated chatbots such as ChatGPT provides a valuable opportunity for improving access to healthcare information. As AI-generated information becomes more prevalent in the medical field, it is important to evaluate its quality and readability and thus its potential use as a tool for patient education. To date, just one study has been published comparing the readability of PEMs generated by ChatGPT-3.5 versus expert-generated materials. The authors found ChatGPT-3.5-generated material on implant-based breast reconstruction to be less readable (grade 10.5) compared to expert-generated material (grade 7.5) [14]. Since then, however, OpenAI has released a superior version of ChatGPT, ChatGPT-4 [15].

In this study, we assessed the capability of ChatGPT-4 to provide adequate patient education. The main aim of this study was to compare the readability and quality of PEMs for TKA produced by ChatGPT-4 and those created by human experts, specifically evaluating their usability for patient education. We seek to determine if ChatGPT-4 can enhance access to high-quality and readable healthcare information to improve patient understanding for TKA.

This article was previously posted to the Research Square preprint server on November 14, 2024.

## Materials and methods

As ChatGPT is a publicly accessible platform, no permissions were required for utilizing the information generated in this study. All data remains confidential and accessible for review to ensure reproducibility. Since the study did not involve human subjects, no ethics committee was formed for review.

We asked ChatGPT-4, a popular large language model by OpenAI, the following prompt five different times: “I am a patient considering a total knee replacement. Provide me information on total knee replacement surgery”. A new chat was opened each time to ensure responses were independent and original. Five human-generated PEMs were gathered from reputable online health resources from the first page of Google using the search phrase “total knee replacement patient” [16-20]. Inclusion criteria included government, hospital, and medical education websites. Output text from ChatGPT-4 and human-generated PEMs were copied into separate standardized text processing documents with grammar/spellcheck disabled for further analysis. Content unrelated to patient education text was omitted from the analysis. This included website hyperlinks, author information, copyright notices, and contact information.

The materials were analyzed using the Flesch-Kincaid Grade Level (FKGL) and Flesch Reading Ease (FRE) for readability. FKGL correlates to the required school grade level of education to understand a text. FRE assesses the readability level on a scale from 0 to 100, where lower scores indicate a higher level of reading difficulty [21]. FRE and FKGL are two of the most popular and validated measures of readability used today; hence, they were selected for use in this study [22]. These measures have been used in a number of studies on the readability of PEMs [23-25].

The reliability and quality of information were assessed using the validated DISCERN criteria. The DISCERN tool is a widely used method for assessing the quality of health-related videos and information. Originally developed by a team from Oxford University and the British Library, it was intended for use by patients and the general public. The DISCERN questionnaire includes 16 items divided into three parts: the first eight questions evaluate reliability, the next seven assess treatment information, and the final question addresses overall quality. Higher total scores reflect better quality. This tool has been applied in previous studies reviewing YouTube content on rotator cuff tears and repairs. Based on total scores, materials are rated as follows: very poor (16-26), poor (27-38), moderate (39-50), good (51-62), and excellent (63-75) [26].

The analysis was carried out by two orthopedic residents with experience in TKA (KL and MM). To account for potential interobserver differences, we used an average consensus method: both reviewers scored independently, and the mean of their scores was used for analysis. Results for FKGL, FRE, and DISCERN were entered into the statistical software Jamovi 1.6.23. Student’s t-test was performed to compare the mean scores obtained from ChatGPT-4 and human-generated PEMs. A significance level of 0.05 was chosen to determine statistical significance.

## Results

There were five human-generated and five AI-generated PEMs. In assessing PEMs for readability using the FKGL, significant differences emerged between human-generated content and outputs from ChatGPT-4. Analysis revealed that ChatGPT-4-generated PEMs had a higher FKGL score of 13.1, whereas human-generated materials registered an FKGL score of 8.52. This difference was statistically significant (p < 0.001). Similarly, FRE was significantly lower for ChatGPT-4 material (33) compared to human-generated materials (58.5) (p < 0.001) (Table 1). There was no statistical difference between DISCERN results (0.506), the reliability, and the quality of information. Individual scores for each PEM are listed in Table 2.

**Table 1:** Descriptive statistics: ChatGPT-4 (AI) vs. human-generated (H) PEMs AI, AI-generated PEM; FKGL, Flesch-Kincaid Grade Level; FRE, Flesch Reading Ease; H, human-generated patient education material; PEM, patient education material

Measure	Source	FKRGL	FKRE	DISCERN
N	H	5	5	5
	AI	5	5	5
Missing	H	0	0	0
	AI	0	0	0
Mean	H	8.52	58.5	57.6
	AI	13.1	33	55.8
Median	H	8.9	56.8	55
	AI	12.7	30.7	57
Standard deviation	H	1.41	6.12	4.56
	AI	0.98	3.84	3.56
Minimum	H	6.8	50.6	53
	AI	12.2	29.7	50
Maximum	H	10.4	65	63
	AI	14.6	37.9	59

**Table 2:** Reading grade level and DISCERN score of PEMs DISCERN score = average between two researchers FKGL, Flesch-Kincaid Grade Level; FRE, Flesch Reading Ease; PEM, patient education material

Source	FKRGL	FKRE	DISCERN
Human-generated 1	6.8	65	63
Human-generated 2	7.5	64.4	55
Human-generated 3	9	56.8	62
Human-generated 4	8.9	55.8	53
Human-generated 5	10.4	50.6	55
ChatGPT-4 PEM 1	12.4	36.4	50
ChatGPT-4 PEM 2	14.6	29.7	58
ChatGPT-4 PEM 3	12.7	30.7	57
ChatGPT-4 PEM 4	12.2	37.9	55
ChatGPT-4 PEM 5	13.5	30.3	59

## Discussion

To our knowledge, this is the first manuscript that explores the use of Chat-GPT4 for PEM for total knee replacement. Low readability indicates difficulty in thoroughly understanding and digesting the presented information, an issue well documented in the medical field [26]. The results of this study elucidate significant discrepancies in readability between human-generated PEMs and those produced by ChatGPT-4. The higher FKGL and lower FRE for ChatGPT-4 suggest its materials are more difficult to read than human-generated PEMs. This indicates that the AI-generated content may be less accessible to the general patient population, which typically benefits from materials written at a sixth-grade reading level or below, in line with recommendations from health literacy experts. Our results align with previous studies, which have shown ChatGPT to produce PEMs at readability levels far greater than the recommended level [14,27-29]. The poorer readability of ChatGPT-4 content underscores the potential for AI-generated materials to inadvertently alienate or challenge readers without a higher level of education, potentially compromising the effectiveness of patient education.

Reassuringly, our analysis found no statistical difference in the quality of information provided by both sources. Both ChatGPT-4 and human-generated PEMs provided a level of quality and reliability that was regarded as “good”. These results are similar to the existing literature with regard to the quality and reliability of ChatGPT-4-generated health information [27-30].

The implications of these findings are multifaceted. First, they call attention to the need for further refinement of AI tools like ChatGPT-4 in the context of health literacy. Developers should consider incorporating health literacy assessments into algorithms that assess and adjust the readability of generated content to match the target audience's reading ability more closely. Collaboration with health communication experts may also be beneficial. Additionally, the findings underscore the importance of human oversight in curating AI-generated PEMs to ensure they meet established readability standards.

Furthermore, our study suggests a promising role for AI in supplementing human efforts in creating PEMs for TKA. With no significant difference in information quality, AI can significantly enhance the breadth and depth of content available to patients. AI integrated into healthcare systems for patient education, particularly for non-English-speaking populations or those with low literacy levels, could help improve the equality of access to healthcare information. However, to fully achieve this potential, the readability of AI-generated materials must be prioritized to ensure accessibility and comprehension by a broader audience.

There were several limitations to this study. This study focused solely on TKA-related materials, limiting its applicability to other medical fields. Reliance on Flesch-Kincaid metrics alone might not capture all nuances of readability and comprehension. Our study did not incorporate the images within the human-generated fact sheets, which have been shown to improve understanding [31]. Factors like cultural relevance and patient-specific comprehension levels were not considered. There may be associated bias by relying solely on ChatGPT-4; future studies could incorporate a range of AI chatbot sources.

## Conclusions

ChatGPT-4 shows promise for its use in producing PEMs for TKA. It has the same quality and reliability as human-generated PEMs. ChatGPT-4 may have the potential to enhance healthcare education for patients on TKA and a variety of other healthcare conditions. However, its current utility is hampered by readability issues. As such, the authors advise against the use of ChatGPT-4 for patient education on TKA. Future advancements in AI should aim to align more closely with the principles of health literacy, ensuring that the information is not only accurate and reliable but also accessible to all patients. Inclusion of visual aids in AI-generated fact sheets may also be beneficial in future studies for more comprehensive analysis. Collaboration between AI developers and healthcare professionals will be crucial in refining these tools to meet the diverse needs of patients effectively, thereby enhancing the quality and efficacy of patient education in healthcare settings.

## Appendices

Human-generated PEM 1 

Total Knee Replacement  

What is a knee replacement? 

A knee replacement is when doctors remove your damaged knee joint and put in a new knee joint. The new joint is artificial, or man-made. 

A knee replacement is an operation that is done in hospital. A knee replacement is also called a knee arthroplasty. 

A knee replacement can help: 

reduce knee pain 

you to be more active 

improve your flexibility 

you to move more freely 

They can help when medicines and other treatments have not been helpful in managing pain. Knee replacements may be total (the whole joint is replaced) or partial (part of your joint is replaced). 

When is a knee replacement needed? 

Knee replacements are usually thought to be a last option to treat severe knee pain that does not go away. 

The most common reason for a knee replacement is osteoarthritis. This condition can lead to ongoing pain. Some people also find their joints are very stiff. Osteoarthritis can impact your quality of life. 

Knee replacement can also be done if someone has a disability. This can happen after other things have been tried to help reduce pain. 

Most people who have a knee replacement are over 60 years of age. If you have a knee replacement when you're under 50 years, you may need another operation in later life. 

How do I prepare for a knee replacement? 

Your doctor will tell you what you need to do to prepare for surgery. You will be asked to fast (not to eat) before your surgery. 

You may also need to stop taking certain types of medicine.  

What happens during a knee replacement? 

In a total knee replacement, the surgeon removes bone from the outside of your knee joint. The surgeon also removes any damaged cartilage. Cartilage is the tissue at the ends of bones that cushions the joints. 

The removed bone and cartilage are replaced with an artificial joint. This man-made joint is made of metal and plastic. 

In a partial knee replacement, only the damaged part of the knee joint is replaced. The healthy parts are left alone. 

Recovering from a knee replacement 

Most people leave hospital 1-4 days after a knee replacement surgery. How quickly you get back to normal depends on many factors, such as your age, general health and fitness. 

You will probably need a cane, crutches or walking frame in the first few weeks. You will also be given an exercise and physiotherapy program. This will help you recover. 

Most people's strength and flexibility slowly recovers over about 12 months. Doing the exercises given to you makes the recovery quicker. 

Possible complications of knee replacement surgery 

Knee replacement surgery is generally thought to be safe and effective. However, complications can include: 

infections 

blood clots in the leg veins 

problems with the new joint 

continued pain 

injury to your nerves or blood vessels during surgery 

After surgery, you need to watch for any signs of a blood clot, such as: 

if your calf becomes red, swollen or painful 

if you become short of breath or have chest pain 

If these happen, go straight to the hospital emergency department. These could be signs of a blood clot in your leg or your lungs. 

Should I have a knee replacement? 

To help you decide whether to have a knee replacement, you can ask your surgeon questions such as: 

What can I do instead of surgery? 

What are the possible problems and how likely are they? 

What can I expect during the recovery period? 

How much improvement is normal after surgery? 

Would a new knee last all my life? 

Are there any alternatives to a knee replacement? 

Alternatives to surgery depend on what's wrong with your knee joint. 

If you have arthritis, other options to try before knee replacement may include: 

non-impact exercise 

losing weight 

physiotherapy 

medicines 

Human-generated PEM 2 

Overview 

Knee comparisons 

Knee replacement surgery replaces parts of injured or worn-out knee joints. The surgery can help ease pain and make the knee work better. During the surgery, damaged bone and cartilage are replaced with parts made of metal and plastic. 

To decide whether a knee replacement is right for you, a surgeon checks your knee's range of motion, stability, and strength. X-rays help show the extent of damage. 

The right artificial joints and surgical techniques for you depend on your age, weight, activity level, knee size and shape, and overall health. 

Products and services 

Why it's done 

The most common reason for knee replacement surgery is to ease pain caused by arthritis. People who need knee replacement surgery usually have problems walking, climbing stairs and getting up out of chairs. 

If only one part of the knee is damaged, surgeons often can replace just that part. If the entire joint needs to be replaced, the ends of the thighbone and shinbone are reshaped and the entire joint resurfaced. These bones are hard tubes that contain a soft center. The ends of the artificial parts are inserted into the softer central part of the bones. 

Ligaments are bands of tissue that help hold joints together. If the knee's ligaments aren't strong enough to hold the joint together by themselves, the surgeon may choose implants that can be connected so they can't come apart. 

Risks 

Knee replacement surgery, like any surgery, carries risks. They include: 

Blood clots. Surgeons typically recommend blood-thinning medications to prevent this risk. The most common location for blood clots is in the leg. But they can travel to the lungs and become deadly. 

Nerve damage. Nerves in the area where the implant is placed can be injured. Nerve damage can cause numbness, weakness and pain. 

Infection. Infection can occur at the incision site or in the deeper tissue. Surgery is sometimes needed to treat an infection. 

The implants used for knee replacements are durable, but they may loosen or become worn over time. If this happens, another surgery may be needed to replace the loose or worn parts. 

How you prepare 

Food and medications 

Your health care team might advise you to stop taking certain medications and dietary supplements before your surgery. You'll likely be instructed not to eat anything after midnight the day of your surgery. 

Prepare for your recovery 

For several weeks after the procedure, you might need to use crutches or a walker, so arrange for them before your surgery. Make sure you have a ride home from the hospital and help with everyday tasks, such as cooking, bathing and doing laundry. 

To make your home safer and easier to navigate during recovery, consider doing the following: 

Create a living space on one floor since climbing stairs can be difficult. 

Install safety bars or a secure handrail in your shower or bath. 

Secure stairway handrails. 

Get a stable chair with a firm seat cushion and back, and a footstool to elevate your leg. 

Arrange for a toilet seat riser with arms if you have a low toilet. 

Get a stable bench or chair for your shower. 

Remove loose rugs and cords. 

What you can expect 

When you check in for your surgery, you'll be asked to remove your clothes and put on a hospital gown. You'll be given either a spinal block, which numbs the lower half of your body, or a general anesthetic, which puts you into a sleep-like state. 

Your surgeon might also inject a numbing medicine around nerves or in and around the joint to help block pain after your surgery. 

During the procedure 

Knee replacement surgery usually takes 1 to 2 hours. To perform the procedure, the surgeon: 

Makes an incision over the knee. 

Removes diseased and damaged bone and cartilage, leaving healthy bone intact. 

Implants the replacement parts into the thighbone, shinbone and kneecap. 

After the procedure 

Knee replacement surgery 

After surgery, you'll rest in a recovery area for a short time. How long you stay in the hospital after surgery depends on your individual needs. Many people can go home the same day. 

The risk of blood clots increases after knee replacement surgery. To prevent this complication, you may need to: 

Move early. You'll be encouraged to sit up and walk with crutches or a walker soon after surgery. 

Apply pressure. Both during and after surgery, you might wear elastic compression stockings or inflatable air sleeves on your lower legs. The air sleeves squeeze and release your legs. That helps keep blood from pooling in the leg veins, reducing the chance that clots will form. 

Take blood thinners. Your surgeon might prescribe an injected or oral blood thinner after surgery. Depending on how soon you walk, how active you are and your overall risk of blood clots, you might need blood thinners for several weeks after surgery. 

You'll also likely be asked to do frequent breathing exercises and gradually increase your activity level. A physical therapist can show you how to exercise your new knee. After you leave the hospital, you'll likely continue physical therapy at home or at a center. 

Results 

For most people, knee replacement provides pain relief, improved mobility and a better quality of life. Most knee replacements can be expected to last at least 15 to 20 years. 

After recovery, you can engage in various low-impact activities, such as walking, swimming, golfing or biking. But you should avoid higher impact activities, such as jogging, and sports that involve contact or jumping. Talk to your health care team about ways to stay active after knee replacement. 

Human-generated PEM 3

Knee Replacement Surgery 

The knee is a hinge joint involving the thigh bone (femur) and the shin bone (tibia). Knee replacement surgery is a technique that removes a diseased knee joint and replaces it with an artificial joint (prosthesis). The most common reason for this operation is severe osteoarthritis, which causes relentless pain, joint deformity and mobility problems. Knee replacement surgery is known as ‘total knee arthroplasty’. 

Conditions that can be treated with knee replacement 

Knee replacement can be used to replace a knee joint affected by a range of conditions including: 

Severe osteoarthritis 

Ligament damage or infection that leads to severe osteoarthritis 

Rheumatoid arthritis 

Haemophilia 

Crystal deposition diseases such as gout and ‘pseudogout’ 

Avascular necrosis - death of bone following loss of blood supply 

Bone dysplasias - disorders of the growth of bone. 

Medical issues to consider 

Prior to the operation, you will need to discuss a range of issues with your doctor or surgeon, including: 

Thorough assessment of your knee joint, which may include x-rays and other imaging techniques. 

Your medical history. If you are elderly, you will need to undergo tests to make sure you are fit for the operation. These tests may include an electrocardiogram and blood tests. 

Inform your doctor about any drugs you may be regularly taking, particularly drugs that affect the blood’s ability to clot such as aspirin or Warfarin. 

Your expectations - you need to understand that although the prosthesis is sophisticated, it can’t replicate the full function of a healthy knee joint. Possible complications of surgery will also be discussed. 

Operation procedure 

The procedure of knee replacement surgery includes: 

You are given antibiotics and blood-thinning drugs about half an hour before the surgery. These drugs help to prevent complications such as infection and blood clots. 

Your leg is cleaned and prepared for surgery. 

You lie on your back on the operating table. 

You are given either a general anaesthetic (which renders you unconscious) or a spinal anaesthetic (which numbs you from the injection site down). 

Mechanical devices to reduce the risk of clots, such as stockings or foot pumps, are used during the operation. 

The incision is up to 30cm long, extending from above your kneecap to below. 

Soft tissue, such as muscle, is moved to expose the knee joint. 

The tibia and femur are cut, and the diseased knee joint is removed. 

Further bone from the tibia and femur may be removed to make sure the prosthetic knee joint sits in the correct position. 

Usually, a special type of glue called bone cement is used to anchor the prosthetic knee to the femur and tibia. 

If needed, the kneecap (patella) is replaced with a prosthetic ‘button’. 

Ligaments and muscles are rearranged. 

A drainage tube is inserted into the wound. 

The incision is closed with stitches or clips. 

The operation can take between two and four hours. 

Immediately after the operation 

After the operation, you can expect: 

Your knee is covered with a dressing and a drainage tube removes excess fluids from the wound. 

You are monitored by nursing staff who regularly check your vital signs (such as blood pressure). 

You are given antibiotics to reduce the risk of infection. 

You are given medications to thin your blood and reduce the risk of clots both during and after the operation. 

Strong pain relief can be given via an epidural or drip. 

You can start eating again on the second day after your operation. 

Nurses encourage you to move your feet and bend your other leg as soon as you can - this helps to reduce the risk of clot formation. 

You are encouraged to walk around on the second day after surgery. 

Physiotherapists show you how to perform knee exercises. 

Occupational therapists advise you on how to best modify your home to make daily life easier during your recovery (for example, the use of safety rails and walking aids). 

Knee replacement surgery without complications usually involves a seven to 10 day hospital stay. 

Your stitches are removed about 10 days after surgery. 

Possible complications 

Some of the possible risks and complications of knee replacement surgery can include: 

Allergic reaction to the anaesthetic 

Wound infection 

Amputation of the leg due to severe wound infection 

Joint dislocation 

The prosthesis breaking or working itself loose 

Temporary or permanent numbness around the incision site 

Paralysis of the foot due to nerve damage 

Lack of blood supply to the leg due to blood vessel damage (this can sometimes lead to amputation) 

Lung infection 

Clots in the veins of the legs 

Circulation difficulties 

Heart attack 

Stroke 

Death. 

Taking care of yourself at home 

Be guided by your doctor or surgeon, but general suggestions include: 

The pain and stiffness take time to ease, so be patient. It may take around three months before you feel fully recovered. 

Keep your wound site clean and dry. 

Avoid smoking - cigarette smoke can increase your risk of lung infections. 

Avoid any sporting activities for at least two months. 

Follow the suggestions given to you by medical staff on how to walk, climb stairs, and get in and out of chairs safely. 

Avoid jumping, jolting the knee joint or kneeling down. 

Use aids to help you around the home - for example, handrails at the bath and toilet, footstools, raised toilet seats, crutches and walking sticks. 

Check your knee carefully for any signs of infection. These can include redness, swelling, warmth or seepage. 

See your doctor or surgeon if you experience anything unusual, such as clicking or popping sounds coming from the knee joint, or a sudden loss of joint control or movement. 

Long-term outlook 

Nine out of 10 patients who have knee replacement surgery experience less pain and greater mobility. You will need to have regular check-ups for the life of your artificial knee and ongoing rehabilitation such as physiotherapy and special exercises. In most cases, the prosthesis can be expected to last around 10 years or so, but excessive wear and tear can reduce its life span. It is particularly important to maintain a healthy weight for your height, as being obese can quickly wear out the prosthesis. You will need to understand that your artificial knee will never function as fully as a healthy one. 

Other forms of treatment 

Without replacement surgery, a severely osteoarthritic knee joint may continue to deteriorate until it is impossible to go about your normal daily activities, such as standing up, walking or getting up from a seated position. Other (less effective) forms of treatment include: 

The use of walking aids, such as frames or walking sticks 

Non-steroidal anti-inflammatory drugs (NSAIDs) 

Corticosteroid injections 

Other surgery, such as osteotomy - an operation in which diseased bone is cut away in an attempt to properly align the malformed joint. 

Human-generated PEM 4

What is a knee replacement surgery? 

Knee replacement, also called knee arthroplasty or total knee replacement, is a surgical procedure to resurface a knee damaged by arthritis. Metal and plastic parts are used to cap the ends of the bones that form the knee joint, along with the kneecap. This surgery may be considered for someone who has severe arthritis or a severe knee injury. 

Various types of arthritis may affect the knee joint. Osteoarthritis, a degenerative joint disease that affects mostly middle-aged and older adults, may cause the breakdown of joint cartilage and adjacent bone in the knees. Rheumatoid arthritis, which causes inflammation of the synovial membrane and results in excessive synovial fluid, can lead to pain and stiffness. Traumatic arthritis, arthritis due to injury, may cause damage to the cartilage of the knee. 

The goal of knee replacement surgery is to resurface the parts of the knee joint that have been damaged and to relieve knee pain that cannot be controlled by other treatments. 

Anatomy of the knee 

Joints are the areas where 2 or more bones meet. Most joints are mobile, allowing the bones to move. Basically, the knee is 2 long leg bones held together by muscles, ligaments, and tendons. Each bone end is covered with a layer of cartilage that absorbs shock and protects the knee. 

There are 2 groups of muscles involved in the knee, including the quadriceps muscles (located on the front of the thighs), which straighten the legs, and the hamstring muscles (located on the back of the thighs), which bend the leg at the knee. 

Tendons are tough cords of connective tissue that connect muscles to bones. Ligaments are elastic bands of tissue that connect bone to bone. Some ligaments of the knee provide stability and protection of the joints, while other ligaments limit forward and backward movement of the tibia (shin bone). 

The knee consists of the following: 

Tibia. This is the shin bone or larger bone of the lower leg. 

Femur. This is the thighbone or upper leg bone. 

Patella. This is the kneecap. 

Cartilage. A type of tissue that covers the surface of a bone at a joint. Cartilage helps reduce the friction of movement within a joint. 

Synovial membrane. A tissue that lines the joint and seals it into a joint capsule. The synovial membrane secretes synovial fluid (a clear, sticky fluid) around the joint to lubricate it. 

Ligament. A type of tough, elastic connective tissue that surrounds the joint to give support and limits the joint's movement. 

Tendon. A type of tough connective tissue that connects muscles to bones and helps to control movement of the joint. 

Meniscus. A curved part of cartilage in the knees and other joints that acts as a shock absorber, increases contact area, and deepens the knee joint. 

Reasons for the procedure 

Knee replacement surgery is a treatment for pain and disability in the knee. The most common condition that results in the need for knee replacement surgery is osteoarthritis. 

Osteoarthritis is characterized by the breakdown of joint cartilage. Damage to the cartilage and bones limits movement and may cause pain. People with severe degenerative joint disease may be unable to do normal activities that involve bending at the knee, such as walking or climbing stairs, because they are painful. The knee may swell or "give-way" because the joint is not stable. 

Other forms of arthritis, such as rheumatoid arthritis and arthritis that results from a knee injury, may also lead to degeneration of the knee joint. In addition, fractures, torn cartilage, and/or torn ligaments may lead to irreversible damage to the knee joint. 

If medical treatments are not satisfactory, knee replacement surgery may be an effective treatment. Some medical treatments for degenerative joint disease may include, but are not limited to, the following: 

Anti-inflammatory medications 

Glucosamine and chondroitin sulfate 

Pain medications 

Limiting painful activities 

Assistive devices for walking (such as a cane) 

Physical therapy 

Cortisone injections into the knee joint 

Viscosupplementation injections (to add lubrication into the joint to make joint movement less painful) 

Weight loss (for obese persons) 

There may be other reasons for your doctor to recommend a knee replacement surgery. 

Risks of the procedure 

As with any surgical procedure, complications can occur. Some possible complications may include, but are not limited to, the following: 

Bleeding 

Infection 

Blood clots in the legs or lungs 

Loosening or wearing out of the prosthesis 

Fracture 

Continued pain or stiffness 

The replacement knee joint may become loose, be dislodged, or may not work the way it was intended. The joint may have to be replaced again in the future. 

Nerves or blood vessels in the area of surgery may be injured, resulting in weakness or numbness. The joint pain may not be relieved by surgery. 

There may be other risks depending on your specific medical condition. Be sure to discuss any concerns with your doctor prior to the procedure. 

Before the procedure 

Your doctor will explain the procedure to you and offer you the opportunity to ask any questions that you might have about the procedure. 

You will be asked to sign a consent form that gives your permission to do the procedure. Read the form carefully and ask questions if something is not clear. 

In addition to a complete medical history, your doctor may perform a complete physical examination to ensure you are in good health before undergoing the procedure. You may undergo blood tests or other diagnostic tests. 

Notify your doctor if you are sensitive to or are allergic to any medications, latex, tape, and anesthetic agents (local and general). 

Notify your doctor of all medications (prescribed and over-the-counter) and herbal supplements that you are taking. 

Notify your doctor if you have a history of bleeding disorders or if you are taking any anticoagulant (blood-thinning) medications, aspirin, or other medications that affect blood clotting. It may be necessary for you to stop these medications prior to the procedure. 

If you are pregnant or suspect that you are pregnant, you should notify your doctor. 

You will be asked to fast for eight hours before the procedure, generally after midnight. 

You may receive a sedative prior to the procedure to help you relax. 

You may meet with a physical therapist prior to your surgery to discuss rehabilitation. 

Arrange for someone to help around the house for a week or two after you are discharged from the hospital. 

Based on your medical condition, your doctor may request other specific preparation. 

During the procedure 

Knee replacement requires a stay in a hospital. Procedures may vary depending on your condition and your doctor's practices. 

Knee replacement surgery is most often performed while you are asleep under general anesthesia. Your anesthesiologist will discuss this with you in advance. 

Generally, knee replacement surgery follows this process: 

You will be asked to remove clothing and will be given a gown to wear. 

An intravenous (IV) line may be started in your arm or hand. 

You will be positioned on the operating table. 

A urinary catheter may be inserted. 

If there is excessive hair at the surgical site, it may be clipped off. 

The anesthesiologist will continuously monitor your heart rate, blood pressure, breathing, and blood oxygen level during the surgery. 

The skin over the surgical site will be cleansed with an antiseptic solution. 

The doctor will make an incision in the knee area. 

The doctor will remove the damaged surfaces of the knee joint and resurface the knee joint with the prosthesis. The knee prosthesis is made up of metal and plastic. The most common type of artificial knee prosthesis is a cemented prosthesis. Uncemented prostheses are not commonly used anymore. A cemented prosthesis attaches to the bone with surgical cement. An uncemented prosthesis attaches to the bone with a porous surface onto which the bone grows to attach to the prosthesis. Sometimes, a combination of the 2 types is used to replace a knee. The prosthesis is generally comprised of 3 components: the tibial component (to resurface the top of the tibia, or shin bone); the femoral [thigh bone] component (to resurface the end of the thighbone; and the patellar component (to resurface the bottom of the kneecap that rubs against the thighbone). 

The incision will be closed with stitches or surgical staples. 

A drain may be placed in the incision site to remove fluid. 

A sterile bandage or dressing will be applied. 

After the procedure 

In the hospital 

After the surgery you will be taken to the recovery room for observation. Once your blood pressure, pulse, and breathing are stable and you are alert, you will be taken to your hospital room. Knee replacement surgery usually requires an in-hospital stay of several days. 

It is important to begin moving the new joint after surgery. A physical therapist will meet with you soon after your surgery and plan an exercise program for you. A continuous passive motion (CPM) machine may be used to begin the physical therapy. This machine moves your new knee joint through its range of motion while you are resting in bed. Your pain will be controlled with medication so that you can participate in the exercise. You will be given an exercise plan to follow both in the hospital and after discharge. 

You will be discharged home or to a rehabilitation center. In either case, your doctor will arrange for continuation of physical therapy until you regain muscle strength and good range of motion. 

At home 

Once you are home, it is important to keep the surgical area clean and dry. Your doctor will give you specific bathing instructions. The stitches or surgical staples will be removed during a follow-up office visit. 

To help reduce swelling, you may be asked to elevate your leg or apply ice to the knee. 

Take a pain reliever for soreness as recommended by your doctor. Aspirin or certain other pain medications may increase the chance of bleeding. Be sure to take only recommended medications. 

Notify your doctor to report any of the following: 

Fever 

Redness, swelling, bleeding, or other drainage from the incision site 

Increased pain around the incision site 

You may resume your normal diet unless your doctor advises you differently. 

You should not drive until your doctor tells you to. Other activity restrictions may apply. Full recovery from the surgery may take several months. 

It is important that you avoid falls after your knee replacement surgery, because a fall can result in damage to the new joint. Your therapist may recommend an assistive device (cane or walker) to help you walk until your strength and balance improve. 

Making certain modifications to your home may help you during your recovery. These modifications include, but are not limited to, the following: 

Proper handrails along all stairs 

Safety handrails in the shower or bath 

Shower bench or chair 

Raised toilet seat 

Long-handled sponge and shower hose 

Dressing stick 

Sock aid 

Long-handled shoe horn 

Reaching stick to grab objects 

Removing loose carpets and electrical cords that may cause you to trip 

Avoiding stair-climbing until recommended by your doctor 

Your doctor may give you additional or alternate instructions after the procedure, depending on your particular situation. 

Human-generated PEM 5

What is Knee Arthritis? 

The bones in a normal knee are lined with a smooth layer of cartilage. This cartilage allows the adjacent surfaces in the knee to glide smoothly and painlessly against each other. 

In the process of arthritis the cartilage is worn away (degenerates) with the end result of “bone rubbing on bone”. This process is painful and can result in inflammation of the knee joint. With progression of the disease the knee becomes increasingly painful, stiff and swollen, and may result in a deformity (bow legged or knock knee). 

What is a Total Knee Replacement? 

A total knee replacement is a prosthesis that is used to replace a knee joint that is affected by arthritis. It consistents of several components: 

An upper metal femoral component that is shaped and sized to fit to the contour of the end of the femur bone 

A metal tibial component which is flat and has a small stem attached to the undersurface of it. It sits on top of the tibia on the opposing side of the knee joint. Both metal femoral and tibial components are made of metal alloys, comprising of cobalt-chrome or titanium. They are both fixed to the bone with a special polymer called “bone cement” 

A plastic insert (“polyethylene insert”)which locks in to the tibial component sitting in it’s upper surface. The plastic liner is the bearing for which the femoral component moves against. The plastic is made of a special polymer call polyethylene which has been carefully manufactured to allow it last a long time with out wearing out. However, even with the latest manufacturing techniques, this still is similar to your articular cartilage in that it has a limited life span, and excessive forces are placed on it, it will wear out more quickly 

A patellar button which resurfaces the back of your knee cap. This is also made of polyethylene. It is not crucial that this is replaced all the time, and it is at the discretion of your surgeon on whether it is in your best interests to have this performed as well 

When is a Total Knee Replacement helpful? 

The most important reason for surgery is pain that is interfering with your quality of life and is not adequately controlled by other means (medication, injections, physiotherapy, activity modifications etc). This is a very individual decision and depends on your social requirements and activity demands. 

What about minimally invasive surgery (MIS)? 

Depending on the size of your knee, the degree of arthritis and deformity, and the amount of preoperative stiffness, you may be a candidate for MIS. This technique minimises surgical trauma to the quadriceps tendon and allows for faster recovery of strength, improved mobility in the first few weeks and sometimes less pain. Your surgeon will discuss this option with you during your initial consultation. 

Procedures 

What happens on the day of your operation? 

Undergoing a total knee replacement is a major operation. You will require specific tests (blood tests, ECG and other x-rays) and possible review by other specialists to ensure you are in optimal condition prior to your surgery. If you have a pre-existing condition (eg: heart disease or diabetes) you will need to be given the “all clear” by your treating physician before surgery. 

Your anaesthetist will explain to you the various options with regards to anaesthetic during the operation and the anaesthetic most suitable for you. In addition, your anaesthetist will discuss the most effective post-operative pain relief for you. 

What happens on the day of your operation? 

You will be admitted to hospital usually the day of your surgery. Your surgeon will visit you to answer any other questions you may have regarding the surgery and also to mark the affected knee with an ink pen. 

After your anaesthetic has been administered a tourniquet will be applied to your upper thigh and your leg will be painted with antiseptic solution. A routine draping will be performed with sterile sheets to allow exposure only of the knee. 

A vertical incision is made on the front of your knee to allow access to the knee joint itself. The arthritic areas of your knee joint are removed and the bones are fashioned to allow placement of the knee prosthesis (comprising a metal femoral and tibial component) and straighten the leg as most people have some deformity (eg knock knees or bow legs). Bone cement is used to help stabilise the prosthesis to the bone. A plastic (polyethylene) insert is placed between the femoral and tibial components and acts as an articulating shock absorbing surface. The decision to resurface the patella (knee cap) will be dependant on its degree of arthritic involvement. 

Before completion of the operation, the knee is checked for stability, alignment and degree of motion. Often, a small drain will be left in the knee for 24 hours after the surgery to remove unwanted blood from the knee. Dissolving stitches are used to close the wound, and the knee is then wrapped in a well-padded sterile bandage. 

After your operation 

You will wake up in the recovery ward where you will be closely monitored until you are ready to return to your ward. Here you will continue to be observed until you are fully awake. 

You will spend the first post-operative day in bed. A physiotherapist will visit you to give you breathing exercises for your chest as well as exercises for your leg that can be performed whilst in bed. An x-ray of your knee will be taken the day after surgery to confirm optimal placement of the prosthesis. 

Blood thinning medications will be administered daily to reduce the risk of blood clots (DVT) forming in your legs. In addition you will be given special stockings (TEDS) to wear on both of your legs. For the first 24 hours after the operation an inflatable sleeve will intermittently compress your calves to prevent stagnation of blood flow. You will receive intravenous antibiotics for 24 hours after the operation to decrease the risk of infection. 

The dressings will be changed 48 hours after the operation. Your knee will be swollen and may have areas of bruising. This is normal. You will be under the daily supervision of a physiotherapist until you are discharged from hospital. The aims of these visits are to optimise motion in the knee, regain ambulation, improve muscle strength and control knee swelling (please refer to education booklet on Total Knee Replacement Rehabilitation). 

Once you are mobilising safely, have regained appropriate motion in the knee and your pain is controlled by tablets you will be discharged home. Some patients require further in-patient care and are transferred to a rehabilitation unit. 

What happens after you go home? 

Your rehabilitation will continue after you leave hospital to initially maintain and then improve on what you have already achieved. This will take the form of a home exercise program and outpatient physiotherapy visits, which usually lasts 3-6 months. 

An appointment will be made to see your surgeon at 6 weeks after your operation. The goals of your knee replacement surgery are to be walking independently, be able to bend your knee to 100-120 degrees and be pain free. 

ChatGPT-4 PEM 1

Total knee replacement, also known as total knee arthroplasty, is a surgical procedure aimed at relieving pain and restoring function in severely diseased knee joints. This surgery involves cutting away damaged bone and cartilage from your thighbone, shinbone, and kneecap and replacing it with an artificial joint (prosthesis) made of metal alloys, high-grade plastics, and polymers. Here's an overview of key aspects related to total knee replacement:

Reasons for Knee Replacement

Osteoarthritis: This is the most common reason, where the knee cartilage deteriorates with age.

Rheumatoid arthritis: Causes inflammation and can destroy knee cartilage.

Post-traumatic arthritis: Arthritis due to a severe knee injury.

Knee deformity: With severe bowing in or out of the knee.

Preparing for Surgery

Preparation might involve several preoperative assessments, including blood tests, medical evaluations, and a knee X-ray. Patients may also be asked to stop taking certain medications and to undergo physical therapy to strengthen the knee before surgery.

The Procedure

The surgery generally lasts about one to two hours. The surgeon removes the damaged surfaces of the knee joint and replaces them with the prosthesis. This is a highly technical procedure that requires precise alignment for the artificial joint to function correctly.

Risks and Complications

Like any major surgery, knee replacement comes with risks, such as:

Infection

Blood clots

Implant problems (loosening or wear)

Nerve and blood vessel injury

Stroke or heart attack during or after surgery

Recovery

Recovery involves hospital stay, typically for a few days, followed by several weeks of physical therapy. Most people can resume normal daily activities within 3 to 6 weeks, although a full recovery might take 6 to 12 months. It's crucial to follow a physical therapy routine to regain strength and mobility and to ensure the success of the surgery.

Longevity of Knee Replacements

Modern knee replacements can last more than 20 years. However, their longevity depends on various factors, including the patient's activity level, weight, and overall health.

Lifestyle Changes and Follow-up Care

Patients are advised to avoid high-impact activities that can put excessive stress on the knee joint, such as jogging, jumping, or high-impact sports. Low-impact activities, such as swimming, walking, and biking, are encouraged.

It's important to have realistic expectations and to discuss with your healthcare provider about the outcomes. While total knee replacement can significantly reduce pain and improve quality of life, the artificial knee might not have the same range of motion as a natural knee. Consulting with an orthopedic surgeon can help determine if this procedure is the right option for you, considering your medical history, knee condition, and lifestyle.

ChatGPT-4 PEM 2

Total knee replacement, also known as knee arthroplasty, is a surgical procedure that involves replacing the damaged or diseased parts of the knee joint with artificial parts. This surgery is considered when knee pain and dysfunction become severe and have not responded well to other treatments such as medications, physical therapy, or less invasive surgical procedures. Here's an overview of the key aspects of total knee replacement surgery:

Indications for Surgery

Total knee replacement is typically recommended for individuals with severe arthritis of the knee (such as osteoarthritis, rheumatoid arthritis, or post-traumatic arthritis) who experience significant pain, stiffness, and impaired function that interfere with daily activities and quality of life.

The Procedure

The surgery involves the removal of the damaged surfaces of the knee joint-the ends of the femur (thigh bone) and tibia (shin bone)-and sometimes the underside of the patella (kneecap). These surfaces are then replaced with artificial components made of metal and plastic. The metal components form the new surfaces of the joint, and a plastic spacer is placed between them to provide a smooth gliding surface.

Types of Knee Prostheses

There are various types of knee prostheses available, including:

Fixed-bearing prostheses: These are the most commonly used type, where the plastic cushion is fixed to the metal component.

Mobile-bearing prostheses: These allow a bit more movement, potentially offering a more natural range of motion for some patients.

Medial pivot prostheses: Designed to more closely mimic the knee's natural movement.

Risks and Complications

Like all major surgical procedures, total knee replacement carries risks, such as infection, blood clots, implant wear and loosening, and the need for revision surgery in the future. However, modern surgical techniques and post-operative care strategies have significantly reduced these risks.

Recovery and Rehabilitation

Recovery involves a stay in the hospital for a few days, followed by several weeks of rehabilitation. Physical therapy is crucial to regain strength and mobility in the knee. Most people can resume normal daily activities within 3 to 6 weeks, although full recovery and maximal improvement may take up to a year.

Outcomes

The majority of patients experience significant pain relief and improvement in knee function and quality of life after knee replacement surgery. Modern knee implants can last 15 to 20 years or more, depending on the patient's activity level and other factors.

Making the Decision

The decision to undergo total knee replacement should be made after a thorough discussion with an orthopedic surgeon, taking into account your individual health status, the extent of your knee problems, and your expectations from the surgery.

It's also beneficial to consider the surgery's timing, potential benefits, and the commitment required for post-operative rehabilitation. Consulting with a healthcare provider will help you understand how the surgery fits into your overall treatment plan and life goals.

ChatGPT-4 PEM 3

Total knee replacement, also known as knee arthroplasty, is a surgical procedure that replaces a knee damaged by arthritis or injury with artificial components. The goal is to relieve pain, restore function, and improve quality of life. Here's a comprehensive overview:

Indications

Total knee replacement is considered when conservative treatments (like medication, physical therapy, and lifestyle changes) have failed to provide relief from symptoms such as:

·         Severe knee pain or stiffness that interferes with everyday activities, including walking, climbing stairs, and getting in and out of chairs.

·         Moderate to severe pain while resting, which may affect sleep.

·         Chronic knee inflammation and swelling that doesn't improve with rest or medications.

·         Knee deformity-a bowing in or out of your knee.

·         Failure to substantially improve with other treatments such as anti-inflammatory medications, cortisone injections, lubricating injections, physical therapy, or other surgeries.

Procedure

The surgery involves removing damaged bone and cartilage from your thighbone, shinbone, and kneecap, and replacing it with an artificial joint made of metal alloys, high-grade plastics, and polymers. It is performed under general anesthesia or spinal/epidural anesthesia and typically requires a hospital stay.

Types of Knee Prostheses

There are different types of knee prostheses, including:

·         Fixed-bearing prostheses: The most common type, suitable for most patients.

·         Mobile-bearing prostheses: Allow more rotation and might be chosen for younger, more active patients.

·         Medial pivot prostheses: Designed to more closely mimic the knee's natural movement.

·         Custom-made prostheses: Tailored to fit the unique shape of your knee.

Recovery and Rehabilitation

Recovery involves hospital stay for a few days post-surgery, followed by several weeks of physical therapy. Rehabilitation is crucial for recovery and can last for months. The goals are to reduce swelling, restore knee movement, and strengthen the leg. Most people can resume normal daily activities within 3 to 6 weeks, although complete recovery might take 6 to 12 months.

Risks and Complications

As with any major surgery, knee replacement comes with risks, such as:

·         Infection

·         Blood clots

·         Implant problems (loosening or wear)

·         Nerve and blood vessel damage

·         Stiffness

·         Continued pain

Outcomes

Most knee replacements are successful, providing significant pain relief, improved mobility, and a better quality of life. Artificial knees can last 15 years or more, but longevity depends on activity level, weight, and other factors.

Decision Making

Deciding to undergo knee replacement involves weighing the benefits against the potential risks and complications. Discuss thoroughly with your orthopedic surgeon, considering your health, activity level, and expectations. They can provide personalized advice based on your condition.

It's also beneficial to explore patient education resources, support groups, and possibly seek a second opinion to make an informed decision.

This overview gives a general understanding of total knee replacement surgery. However, individual cases vary, so specific questions and concerns should be addressed with your healthcare provider.

ChatGPT-4 PEM 4

Total knee replacement surgery, also known as knee arthroplasty, is a procedure to replace the weight-bearing surfaces of the knee joint to relieve pain and disability, most commonly caused by osteoarthritis. Other conditions that cause knee damage, such as rheumatoid arthritis and psoriatic arthritis, may also be reasons for considering this surgery. Here’s an overview of the essential aspects of total knee replacement:

Indications for Surgery

·         Severe knee pain or stiffness that limits everyday activities, including walking, climbing stairs, and getting in and out of chairs.

·         Moderate or severe knee pain while resting, either day or night.

·         Chronic knee inflammation and swelling that doesn’t improve with rest or medications.

·         Knee deformity - a bowing in or out of your knee.

·         Failure to substantially improve with other treatments such as anti-inflammatory medications, cortisone injections, lubricating injections, physical therapy, or other surgeries.

The Procedure

The surgery involves removing damaged cartilage and bone from the surface of your knee joint and replacing them with an artificial joint made of metal alloys, high-grade plastics, and polymers. It is performed under general anesthesia or spinal/epidural anesthesia.

Types of Knee Prostheses

·         Metal on Plastic: The most common type, where the metal femoral component rides on a plastic (polyethylene) tibial component.

·         Ceramic on Plastic: Involves a ceramic femoral component and a plastic tibial component.

·         Ceramic on Ceramic: Both femoral and tibial components are ceramic. They are very smooth and wear very little.

·         Metal on Metal: Less commonly used due to concerns about metal ions in the bloodstream.

Recovery and Rehabilitation

·         Initial recovery typically involves a stay in the hospital for a few days.

·         Physical therapy starts soon after surgery to aid recovery, which can last from a few months to a year.

·         Most people can resume their normal daily activities within 6 weeks, and drive within 3 to 6 weeks.

·         Full knee strength and motion range recovery can take up to a year.

Risks and Complications

·         As with any major surgery, there are risks of complications such as infection, blood clots, nerve damage, and blood vessel injury.

·         There’s also a risk of prosthesis problems, such as loosening or malalignment.

Longevity of the Prosthesis

·         Modern knee replacements are designed to last many years. Most knee replacements last 15-20 years, depending on the patient's activity level, weight, and other factors.

Decision Making

Deciding to undergo knee replacement surgery should be a collaborative process between you, your family, your primary care physician, and your orthopedic surgeon. Factors to consider include the extent of your knee pain, how it affects your life, and your expectations from the surgery.

It’s important to have a thorough discussion with your healthcare provider about the benefits and risks of the surgery, what to expect during recovery, and how to maximize the life of your knee replacement.

ChatGPT-4 PEM 5

Total knee replacement (TKR), also known as knee arthroplasty, is a surgical procedure often recommended for patients with severe knee pain and disability caused by damage or diseases such as osteoarthritis, rheumatoid arthritis, or post-traumatic arthritis. The surgery involves replacing the damaged or diseased parts of the knee joint with artificial parts, called prostheses or implants. Here's an overview of what you need to know about total knee replacement surgery:

Understanding the Surgery

Purpose: To relieve pain, improve knee function, and enhance quality of life by resurfacing the parts of the knee joint that have been damaged.

Procedure: The surgeon removes damaged bone and cartilage from the surface of your knee joint and replaces them with an artificial joint made from metal alloys, high-grade plastics, and polymers.

Preparing for Surgery

Pre-surgical Evaluation: Involves thorough medical evaluation, X-rays, and possibly MRIs to determine if you're a good candidate for the surgery.

Preparation: Might include blood tests, medication adjustments, and physical evaluations. Patients are often advised to stop smoking, manage chronic conditions, and may be given pre-surgical exercises.

The Surgical Process

Anesthesia: General anesthesia or spinal/epidural anesthesia may be used.

Incision: A cut is made in the front of the knee to expose the knee joint.

Resurfacing: Damaged surfaces of the knee joint are removed and replaced with the prosthesis. The prosthesis is designed to replicate the knee's natural movement.

Duration: The surgery usually takes 1 to 2 hours.

Recovery and Rehabilitation

Hospital Stay: Patients typically stay in the hospital for a few days post-surgery.

Rehabilitation: Begins almost immediately after surgery with the goal of strengthening the knee and restoring movement. Physical therapy is a crucial part of the recovery process.

Long-term Care: Includes exercises to maintain knee strength and flexibility, avoiding high-impact activities to prolong the life of the artificial knee.

Risks and Complications

Like all surgeries, total knee replacement comes with risks, including infection, blood clots, implant wear and tear, and the need for revision surgery in the future.

Outcomes

Success Rate: High, with many patients experiencing significant pain relief and improved mobility.

Implant Longevity: Modern knee implants can last 15-20 years or more, depending on the patient's activity level and weight.

Making Your Decision

Deciding to proceed with a total knee replacement involves weighing the potential benefits against the risks and considering your own quality of life and goals. Discuss thoroughly with your orthopedic surgeon, who can provide personalized advice based on your health status, activity level, and expectations.

Conclusion

Total knee replacement surgery can be a life-changing procedure for those suffering from severe knee problems. With proper preparation, successful surgery, and dedicated rehabilitation, many patients are able to return to their daily activities with less pain and improved function.
